# Single Crystal Investigations Unravel the Magnetic Anisotropy of the “Square-In Square” Cr_4_Dy_4_ SMM Coordination Cluster

**DOI:** 10.3389/fchem.2019.00006

**Published:** 2019-01-24

**Authors:** Mauro Perfetti, Julia Rinck, Giuseppe Cucinotta, Christopher E. Anson, Xuejun Gong, Liviu Ungur, Liviu Chibotaru, Marie-Emmanuelle Boulon, Annie K. Powell, Roberta Sessoli

**Affiliations:** ^1^Laboratory of Molecular Magnetism, Università Degli Studi di Firenze, Sesto Fiorentino, Italy; ^2^Institute of Functional Interfaces, Karlsruhe Institute of Technology, Eggenstein-Leopoldshafen, Germany; ^3^Institut für Anorganische Chemie, KIT, Karlsruhe, Germany; ^4^Department of Chemistry, Katholische Universität Leuven, Leuven, Belgium; ^5^Institut für Nanotechnologie, KIT, Eggenstein-Leopoldshafen, Germany

**Keywords:** lanthanides, transition metals, 3d/4f coordination clusters, single crystal magnetometry, torque magnetometry, magnetic anisotropy

## Abstract

In the search for new single molecule magnets (SMM), i.e., molecular systems that can retain their magnetization without the need to apply an external magnetic field, a successful strategy is to associate 3*d* and 4*f* ions to form molecular coordination clusters. In order to efficiently design such systems, it is necessary to chemically project both the magnetic building blocks and the resultant interaction before the synthesis. Lanthanide ions can provide the required easy axis magnetic anisotropy that hampers magnetization reversal. In the rare examples of 3*d/*4*f* SMMs containing Cr^III^ ions, the latter turn out to act as quasi-isotropic anchors which can also interact via 3*d*-4*f* coupling to neighbouring Ln centres. This has been demonstrated in cases where the intramolecular exchange interactions mediated by Cr^III^ ions effectively reduce the efficiency of tunnelling without applied magnetic field. However, describing such high nuclearity systems remains challenging, from both experimental and theoretical perspectives, because the overall behaviour of the molecular cluster is heavily affected by the orientation of the individual anisotropy axes. These are in general non-collinear to each other. In this article, we combine single crystal SQUID and torque magnetometry studies of the octanuclear [Cr_4_Dy_4_(μ_3_-OH)_4_(μ-N_3_)_4_(mdea)_4_(piv)_8_]·3CH_2_Cl_2_ single molecule magnet (piv=pivalate and mdea=*N*-methyldiethanol amine). These experiments allowed us to probe the magnetic anisotropy of this complex which displays slow magnetization dynamics due to the peculiar arrangement of the easy-axis anisotropy on the Dy sites. New *ab initio* calculations considering the entire cluster are in agreement with our experimental results.

## Introduction

In the search for innovative solutions for data storage and manipulation at the nanoscopic scale, magnetic molecules like the so-called single molecule magnets (SMM) could play a predominant role. They could store information (Caneschi et al., 1993; Sessoli et al., 1993; Thomas et al., 1996), and be used for computing (Leuenberger and Loss, 2001; Affronte et al., 2007; Lehmann et al., 2007). Indeed, the electronic spin carried by a molecule is both addressable, on a surface for example (Mannini et al., 2009, 2011; Bhandary et al., 2011), and manipulable using different techniques, two pertinent characteristics for quantum computing (Ardavan et al., 2007; Boulon et al., 2017; Godfrin et al., 2017a,b) or for spintronics (Bogani and Wernsdorfer, 2008; Perrin et al., 2015; Coronado and Yamashita, 2016). The design and the elaboration of functional molecules is however challenging and continue to stimulate the community of chemists and physicists, from both experimental and theoretical points of view. Bringing molecules inside devices requires a deeper understanding of the relevant properties, and one of them is the magnetic anisotropy having led to the development of experimental (Cornia et al., 2001; Cucinotta et al., 2012; Boulon et al., 2013a,b; Perfetti et al., 2014; Meng et al., 2016) and theoretical (Karlström et al., 2003; Aquilante et al., 2010) studies in the past years. There is no doubt that complementary approaches are crucial for improving the properties of molecules, as recently demonstrated with the record of the highest temperature for a SMM (Guo et al., 2018).

The chemistry of complex polynuclear lanthanide systems is constantly developing and, at the current stage, allows to tune and target specific properties by playing with the ligand field (Sessoli and Powell, 2009; Zhang et al., 2013, 2018; Liu et al., 2018). Another strategy to improve magnetic properties can also be to gather *3d* and *4f* ions into a polynuclear complex (Andruh et al., 2009). The strong spin orbit coupling of lanthanides provides the required easy axis magnetic anisotropy, while transition metals can be used to engineer structures with strong exchange interactions. In this respect, the combination of Dy^III^ and Cr^III^ ions has been proven to be the most successful to achieve remarkable magnetic properties (Rinck et al., 2010; Langley et al., 2013, 2015). However, characterising the magnetic anisotropy of such systems remains a challenge.

**Figure 1:**
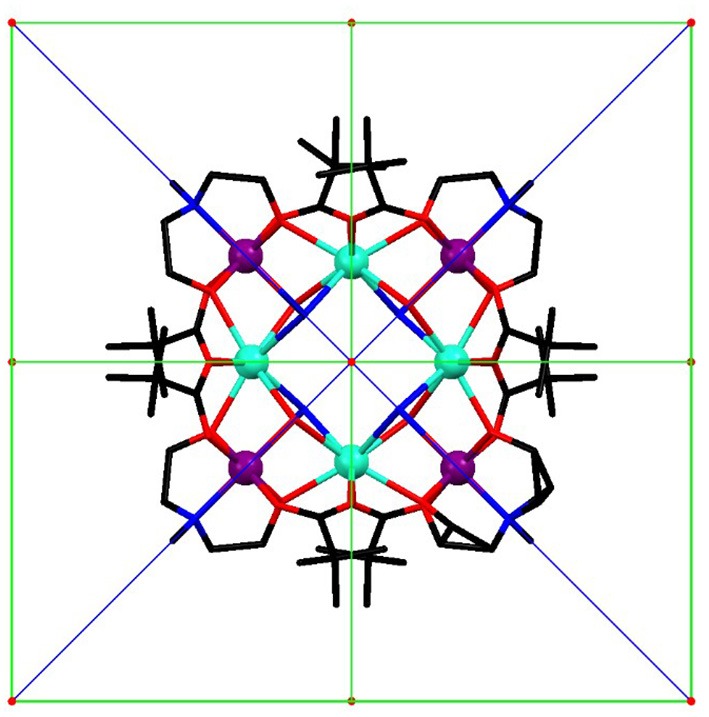
Molecular structure of the [Cr_4_Dy_4_(μ_3_-OH)_4_(μ-N_3_)_4_(mdea)_4_(piv)_8_] complex viewed along the ***c*** axis (turquoise: Dy, purple: Cr, blue: N, red: O, black: C, H are omitted) with symmetry elements: red S_4_ axes, green 2-fold axes, and blue mirrors planes.

With this in mind, we present a combined experimental and theoretical approach to complete the previously reported study on a tetranuclear Dy complex (Rinck et al., 2010). The core of [Cr_4_Dy_4_(μ_3_-OH)_4_(μ-N_3_)_4_(mdea)_4_(piv)_8_]·3CH_2_Cl_2_ is constituted by a perfect square of four Dy^III^ cations. Each pair of adjacent Dy^III^ centres is bridged by a (μ_3_-OH) ligand to a Cr^III^ cation. The four Cr^III^ centres are displaced alternately above and below the Dy_4_ square in the D_2_d site symmetry as represented in Figure 1. In this work, we have used single crystal magnetometry (SCM) which provides a direct measurement of the anisotropy of the magnetic susceptibility complemented by cantilever torque magnetometry (CTM) in order to determine the orientation and magnitude of the magnetic anisotropy of each magnetic ion. Individual anisotropies have previously been deconvoluted both in transition metal clusters and lanthanide polynuclear systems (Rigamonti et al., 2015; Mihalcea et al., 2016) using this very sensitive technique. We have compared here our experimental results with state-of-the-art *ab initio* calculations finding good agreement.

## Materials and Methods

### Synthesis

A crystal of [Cr_4_Dy_4_(μ_3_-OH)_4_(μ-N_3_)_4_(mdea)_4_(piv)_8_]·3CH_2_Cl_2_ was prepared as previously described (Rinck et al., 2010).

### Angular Resolved Magnetometry

Angular resolved susceptibility measurements were performed on a Quantum Design MPMS SQUID magnetometer (Superconducting Quantum Interference Device) using the commercial horizontal rotator from Quantum Design. The single crystal habit was determined by using a single crystal Oxford Xcalibur3 X-Ray diffractometer. The crystal was mounted on a square acetate foil (side ≈ 2 mm) and fixed onto the horizontal rotator using silicon grease. Details about the crystal orientation can be found in Table S1.

### Torque Magnetometry

Torque magnetometry experiments were performed by using a homemade two-legged CuBe cantilever separated by 0.1 mm from a gold plate (Perfetti, 2017). The cantilever was inserted into an Oxford Instruments MAGLAB2000 platform with automated rotation of the cantilever chip in a vertical magnet. The capacitance of the cantilever was detected with an Andeen-Hegerling 2500. An Ultra Precision Capacitance Bridge. Details about the crystal orientation can be found in Table S1.

### *Ab initio* Calculations

*Ab initio* calculations were performed by using MOLCAS 7.8 quantum chemistry package (Aquilante et al., 2010). Each mdea ligand deviates from mirror symmetry by a slight twisting about the Cr-N bond. Two structures of the Cr_4_Dy_4_ compound were therefore considered, which differ in the arrangement of the twist directions of the four ligands of the molecule: **Structure 1** has D_2_ point group symmetry (two Dy sites were calculated *ab initio*) whereas **Structure 2** has an S_4_ point group symmetry (one Dy site was computed *ab initio* since all Dy sites are equivalent). Mononuclear structures containing only one Dy site were built by replacing neighbouring metal sites by their diamagnetic equivalents: Lu was used in place of neighbouring Dy sites while Sc^3+^ ions were employed in place of Cr^3+^ in these calculations. Importantly, the entire ligand framework of the original Cr_4_Dy_4_ molecule was kept unaltered. All atoms were described by ANO-RCC basis sets of VTZP/VDZP quality (Roos et al., 2004, 2005, 2008). The employed basis set contractions are listed in the Supplementary Information. All calculations were of the SA-CASSCF/RASSI kind (Malmqvist et al., 2002; Chibotaru et al., 2008). The active space of the CASSCF method included the 4*f* ^9^ configuration of the Dy site. The spin-orbit coupling was included within RASSI method. All spin sextet states, 128 spin quartet states and 130 spin doublet states arising from the defined active space CAS(9in7) were included in the spin-orbit interaction. In the basis of the obtained spin-orbital multiplets, the *g* tensor, parameters of the crystal field and other related magnetic properties were evaluated within the SINGLE_ANISO module (Aquilante et al., 2010; Chibotaru and Ungur, 2012).

## Results

### Angular Resolved Magnetometry

**Figure 2:**
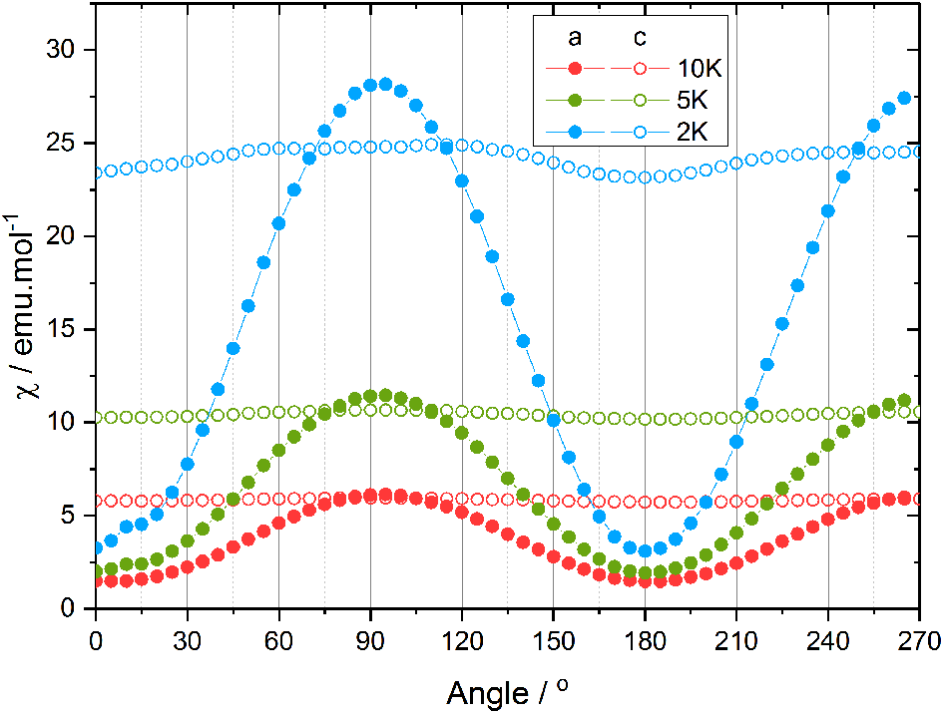
Angular dependence of the magnetic susceptibility at 0.1 T and 2 (blue), 5 (green), and 10 K (red) along ***a*** (filled circles) and ***c*** (open circles).

From the single crystal magnetometry experiment, whose results are reported in Figure 2, it appears that the out-of-plane anisotropy is, as expected for a tetragonal system, more pronounced than the in-plane anisotropy. In rotation along ***c*** (open circles in Figure 2), the resulting in-plane contribution of the anisotropy is investigated whereas for the rotation along ***a***, the magnetic field goes from perpendicular to parallel to the molecular plane (see Table S1). Rotating the sample along ***a*** (filled circles in Figure 2) results in a strong variation of the ratio of the magnetization and the magnetic field *M/B*, assumed at this moderate field to coincide with the susceptibility and reported as χ. This is in agreement with previous theoretical calculations (Figure S1). Indeed, the previously proposed model (Rinck et al., 2010) suggested a strong variation between the in-plane and the out-of-plane magnetic susceptibility. However, the minimum of the magnetic susceptibility is surprisingly measured when the magnetic field is applied along the fourfold axis, and, the maximum when the magnetic field is applied in the (*ab*) plane. Consequently, the rotation along ***c***, during which the magnetic field remains inside the molecular plane is almost constant at the maximum value, the small deviation being attributed to an experimental error of about 2° in the orientation of the crystal for this measurement (see Supplementary Information).

### Single Crystal Magnetization Measurements

**Figure 3:**
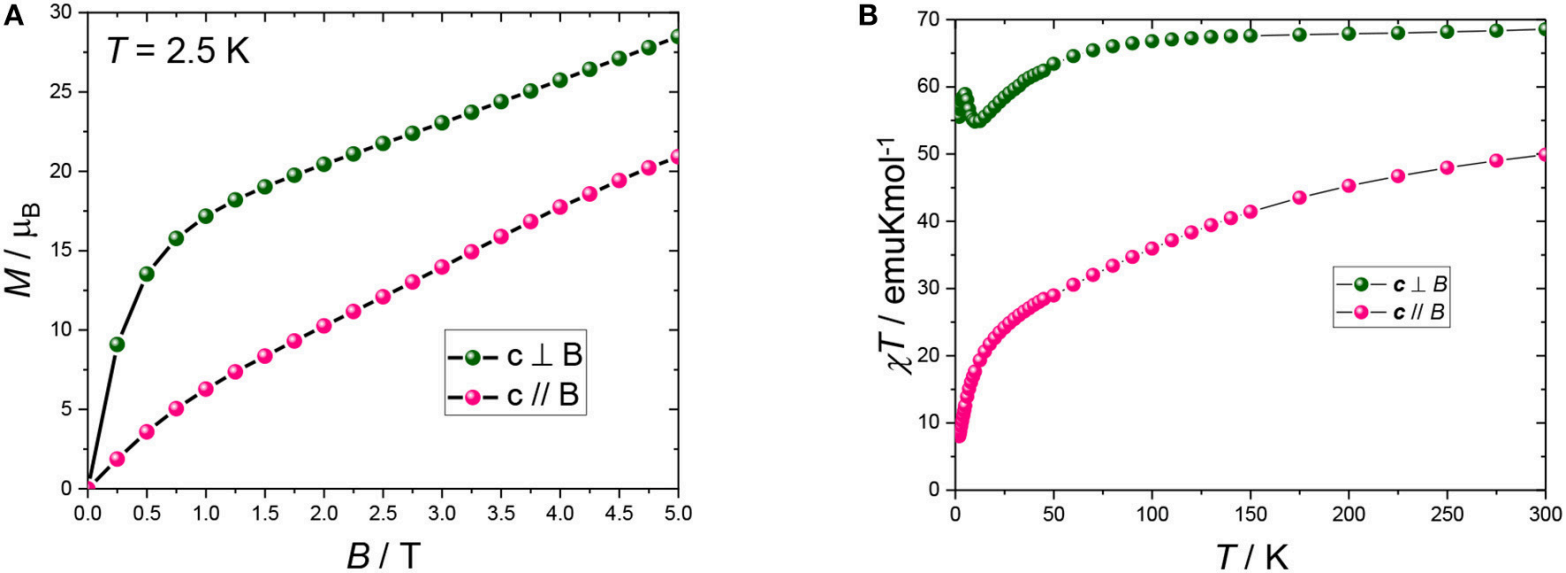
**(A)** Magnetization curves recorded at 2.5 K. **(B)** χ*T* vs. *T* curves recorded at 0.1 T (2–50 K) and 1 T (40–300 K). Pink and green dots represent measurements with the field being parallel and perpendicular to ***c***, respectively. The black lines are a guide to the eye.

Magnetic field dependence of the magnetization and temperature (*T*) dependence of the susceptibility were measured on a single crystal in order to unequivocally determine the orientation of the maximum of the magnetic moment within the crystal. At all fields and temperatures, the magnetic response along the (*ab*) plane is higher than along ***c***. This is in agreement with the angular dependence of the susceptibility measurements. Interestingly, the in-plane χ*T* vs. *T* curve (Figure 3 green dots) exhibits a slight decrease followed by a sharp increase at low temperatures. This behaviour can be attributed to a mixture of effects, namely the depopulation of the CF levels of the Dy^III^ ions and the presence of coupling between the magnetic ions. Conversely, the magnetization curve obtained along ***c*** (Figure 3 pink dots) exhibits a monotonic increase from low to high temperature, with absence of saturation even at room temperature, at difference from what was expected from calculations (Rinck et al., 2010).

If we take the weighted average of the single crystal measurements according to 1/3(χ*T*_||_+ 2 χ*T*_−_) then the obtained room temperature value of 62.3 emuKmol^−1^ is close to that expected for the randomly orientated independent ions of χ*T* = 64.2 emuKmol^−1^.

### Cantilever Torque Magnetometry

**Figure 4:**
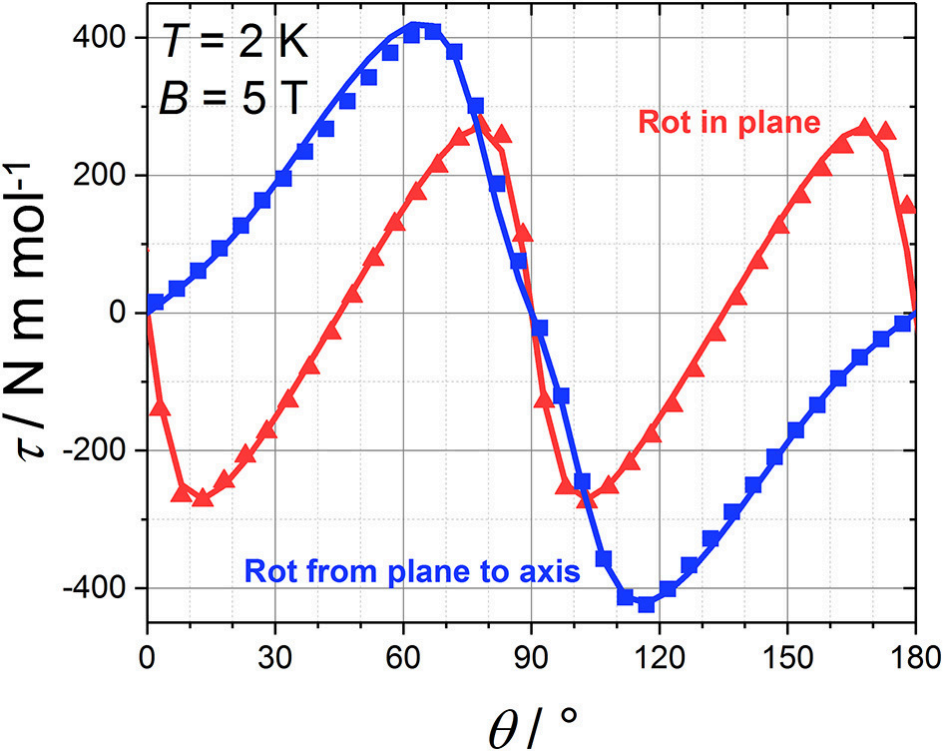
Angular variation of the magnetic torque moment at 2 K and 5 T for both in plane (red) and out of plane (blue) rotation along with the best fit (solid line).

SCM experiments provide the magnetic anisotropy of the system but do not help to disentangle the single centres contributions. The latter are symmetry related but not necessarily coincident if the symmetry of the site is lower than the symmetry of the crystal. The symmetry of the molecule (Figure 1) constrains the main anisotropy axes to lie along the mirror planes of the molecule. Moreover, as the metal ions lie on symmetry elements, mirror planes and 2-fold axes for the Cr^III^ and Dy^III^ ions, respectively, also the individual principal anisotropies show geometrical constraints. In particular, the only free parameter, beyond anisotropy amplitude, is the Euler angle between the *z* magnetic axis of the single centres and the ***c*** crystallographic axis. Cantilever torque magnetometry represents an excellent technique for this purpose. The measurements were performed using high magnetic fields to overwhelm the intramolecular interactions and directly access the single ion contributions. The good alignment of the crystal is proven by the symmetry of the peaks and the position of the zeros in the angular dependence of the torque moment (Figure 4), which in the experimental setup used here is detected along the rotation axis (Table S1). The in-plane rotation exhibits a τ = 0 every 45°, i.e., when the field lies along a principal crystallographic axis and along a mirror plane. The deviation from a perfect sinusoidal curve (steeper/smoother variation of the torque when the field is perpendicular/parallel to the easy axis) is a characteristic feature of torque measurements taken at high fields and is the key to disentangle noncollinear contributions. (Perfetti et al., 2014). Moreover, the out-of-plane rotation shows two significant features: (i) a shoulder at 90° and (ii) the peculiar shape of the torque moment near 0° and 180° with τ increasing less rapidly than expected (Perfetti, 2017). The comparable magnitude between the two rotations indicates that the *z* axes of the lanthanides should be significantly tilted from the ***c*** axis, since the Dy^III^ ions are expected to be the dominant contributors to the anisotropy of the complex.

Due to the intrinsic complexity of this system, our approach to simulate the torque data included the smallest number of parameters able to reproduce the experimental data. A global simulation of all the experimental torque data (Figure 4; Figure S2) was thus obtained using the following spin Hamiltonian, which does not account for the exchange interaction between metal sites:

$$\begin{array}{l} H=\sum_{i=1}^{4}\{\mu_{B}g_{z,\text{Dy}}H_{zi}\cdot {\hat{S}}_{zi}+\mu_{B}g_{xy,Dy}H_{xyi}\cdot {\hat{S}}_{xyi}+\mu_{B}g_{\text{Cr}}H\cdot {\hat{S}}_{i}\\ \;+D_{\text{Cr}}{\hat{S}}_{zi}^{2}\} \end{array}$$

where the summation contains the Zeeman energy (first three terms) and the zero-field splitting (ZFS, fourth term). Coupling terms were neglected due to the high applied fields (*B* ≥ 5T). The Dy^III^ ions were described as *S* = $\frac{1}{2}$ pseudospins with axially anisotropic *g* factors. The Cr^III^ ions were modelled using an isotropic *g* and an axial ZFS term. The individual *z*_*i*_ axes of the four Dy^III^ and Cr^III^ ions are related by the symmetry elements of the molecule. The best agreement with experiments was obtained using the parameters in Table 1.

**Table 1:** Best fit parameters to the torque experiment.

**Ion**	**Spin**	***g_**z**_***	***g_**xy**_***	***D* (cm^**-1**^)**	**$\hat{zc}$(**°**)**
Cr^III^	3/2	2.0 (1)	2.0 (1)	−0.7 (1)	5 (5)
Dy^III^	1/2 (fictitious)	16.5 (2)	2.4 (2)	–	77 (5)

**Figure 5:**
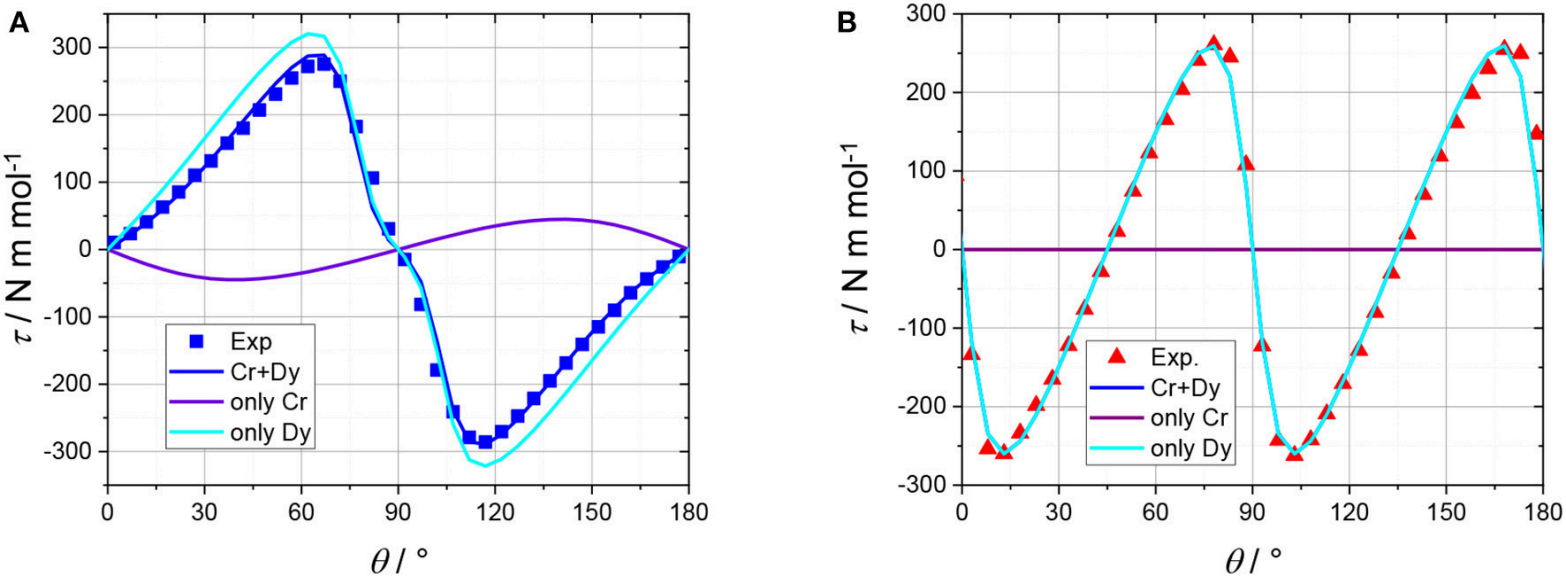
Torque magnetometry data recorded at 2 K and 5 T. At the beginning of the rotation from plane to axis **(A)** the field was oriented along the (*ab*) plane (at 45° from ***a***, see Table S1). At the beginning of the rotation along ***c* (B)** the field was oriented along ***a*** (see Table S1). Symbols are experimental points, the turquoise, and purple lines represent the contributions of the Dy^III^ and Cr^III^ ions, respectively. The solid blue and red lines are the resultant torque of the molecule. The red line on the **(B)** is largely superimposed to the turquoise line since the Cr anisotropy is not significantly contributing.

The shoulder near 90° in the out of plane rotation can only be reproduced if the easy axes of the Dy^III^ ions are very close to the (*ab*) plane (between 75 and 85° from the ***c*** axis, depending on the chosen *g* components). The slope between 0° and 40° (and, by symmetry, between 140° and 180°) can only be reproduced by introducing an axially anisotropic contribution *D* from the Cr^III^ ions (*D* = −0.7 cm^−1^) with the *z* axis at 0–10° from ***c*** (depending on the value of *D*). The correlation between the value of *D* and the angle between the *z* axis of the Cr^III^ ions and ***c*** is intrinsically difficult to disentangle based on the experimental data that we collected. In Figure 5 we reported the contributions of the Dy^III^ and Cr^III^ ions to the torque in both rotations. Interestingly, the Cr^III^ anisotropy does not significantly affect the data in the in-plane rotation, i.e., along ***c***, whose features are unambiguously indicative of the almost in-plane orientation of the Dy^III^ easy axes. The simplification of the model, i.e., neglecting the exchange interaction between the magnetic ions, was necessary for the treatment of the contributions of Dy^III^ and Cr^III^ ions to the total magnetic anisotropy. However, the development of a more complex model, also encompassing interactions and excited states, is ongoing.

### *Ab initio* Calculations

In the previous work published by some of us (Rinck et al., 2010), mononuclear fragments of Dy and Cr units were computed. In the light of the new experimental data, we performed new *ab initio* calculations on the two *full* structures of Cr_4_Dy_4_ molecules without altering the ligand framework for the cluster fragmentation. Table 2 reports the obtained energy spectrum and *g*-tensor of the ground Kramers doublet on the calculated Dy sites and the angle made by the ground main magnetic axis with the ***c*** crystallographic axis for both **Structure 1** and **Structure 2**. On each Dy site there are several excited Kramers doublets with small excitation energy. Therefore, we can expect the lowest of them to be admixed by the Dy^III^-Cr^III^ exchange interaction.

**Table 2:** *Ab initio* calculated low-lying energy splitting (in cm^−1^) of the ground *J* = 15/2 of the Dy sites in the two Cr_4_Dy_4_ structures.

**Structure 1 (D**_****2****_ **symmetry)**		**Structure 2 (S_**4**_ symmetry)**
**Site Dy1**	**Site Dy2**	**Site Dy**
0.0	0.0	0.0
22.3	37.4	29.2
37.8	77.6	58.4
70.1	118.6	91.8
104.4	148.3	123.4
116.3	155.2	135.4
145.1	204.7	176.2
445.8	439.3	442.1
**MAIN VALUES OF THE** ***g*** **tensor IN THE GROUND DOUBLET STATE**		
0.731	0.260	0.414
2.528	0.599	1.103
16.956	19.024	18.563
**ANGLE BETWEEN THE MAIN AXIS** ***g***_***z***_ **AND THE** ***c*** **CRYSTALLOGRAPHIC**		
**AXIS (°)**		
80.29	83.12	83.27

These new computation results, corroborated by the SCM and the CTM experiments, give the main anisotropy axis of the Dy^III^ ions lying much closer to the Dy_4_ plane than predicted by the previously published calculations. The improvement of the output can be explained by the high sensitivity of the *ab initio* results on the cluster fragmentation in the Cr_4_Dy_4_ complexes, as, due to insufficient computational resources, the applied fragmentation was more severe in the previous calculations.

## Discussion

**Figure 6:**
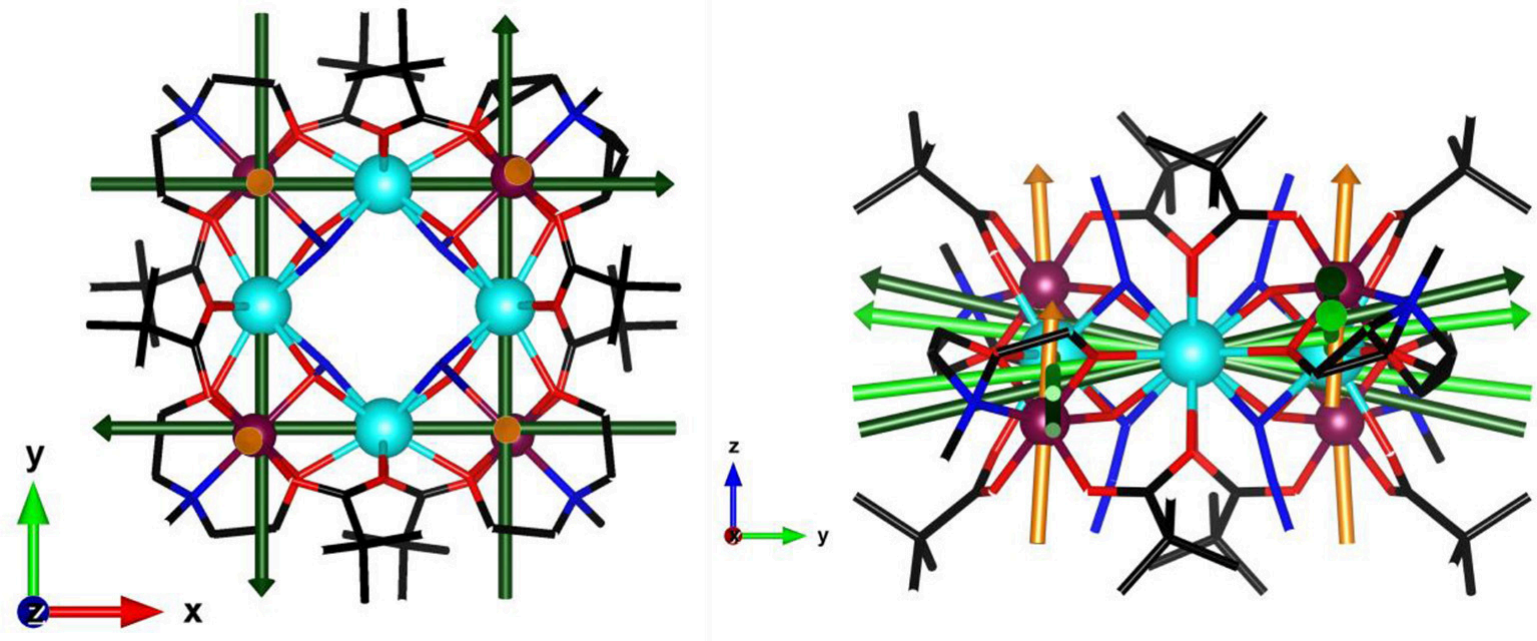
A possible representation of the anisotropy axis for Dy (green, dark: experimental, light green: calculated *ab initio* considering S_4_ symmetry) and Cr (orange, experimental) ions. (turquoise: Dy, purple: Cr, black: C, red: O, blue: N; H are omitted). The Cr anisotropy should be considered as an effective one, able to reproduce the experimental data in the employed simplified model (see text).

In our previous work several theoretical models had been proposed to rationalise the magnetic anisotropy of the Cr_4_Dy_4_ cluster (Rinck et al., 2010). The outcome of the most accurate one suggested that the orientation of the *z* anisotropy axis for the Dy^III^ ions (fictitious *S* = 1/2, *g*_*x*_ = 1.7, *g*_*y*_ = 2.2, *g*_*z*_ = 14.4) stands at an angle of 20.06° from the crystallographic ***c*** axis. Simulating the angular dependence of the magnetic susceptibility with the aforementioned parameters does not reproduce our experimental results and instead presents an opposite phase to what we measure (Figure S1). SCM and CTM results clearly indicate that the easy anisotropy axis of individual Dy^III^ ions is lying close to the molecular plane, thus giving rise to an overall easy plane type behaviour. To shed light on this discrepancy, improved *ab initio* calculations have been performed considering the entire ligand framework of the cluster. The outcome of the improved calculations predicts an orientation of the main anisotropy axis much closer to the (*ab*) plane (80–83° from the crystallographic ***c*** axis, depending on the employed cluster geometry). This now provides excellent agreement with the experiments and constitutes a clear warning: fragmentation of lanthanide clusters might lead to incorrect results. Figure 6 gives a possible representation of the anisotropy axes for all the magnetic ions of the molecule (light green: from *ab initio* calculation and dark green: from experimental results analysis). Note that experiments alone would have left the ambiguity of associating a given easy axis direction to one particular Dy ion, but this ambiguity is resolved by the *ab initio* calculations. The anisotropy of the *g* tensors of the Dy ions extracted from the experiments seems less pronounced compared to the one obtained by the *ab initio* calculation. The physical origin of this discrepancy could be related to interactions not included in our models.

Figure S3 reports the magnetization calculated with the applied magnetic field *B* parallel and perpendicular to ***c*** using a simplified (*S* = 1/2) experimental model. It appears that the low field data at the lowest temperature are poorly reproduced, with experimental data lower than the calculated ones. Interestingly, a better agreement is observed at *T* = 5 K. The advantage of using CTM, with its exceptional sensitivity at high fields, is evident as the agreement with experiments at high fields is better.

However, the exchange interaction between the magnetic ions, which we neglect in our present model, was estimated, in the previous simulations (Rinck et al., 2010), to be one order of magnitude larger than the Cr^III^ ZFS used here [*j*(Cr-Dy) = –(5–10) cm^−1^ vs. *D*(Cr) = −0.7 cm^−1^). Therefore, it could be a driving force for the arrangement of magnetic moments on Cr^III^ ions. Accordingly, in the low-energy exchange states these are expected to lie as close as possible to the plane of the two neighbouring Dy magnetic moments, i.e., close to the (*ab*) plane. The *ab initio* calculations also predict two low-lying excited Kramers doublets on each Dy site, which would be expected to be admixed by the exchange interaction and would contribute to the field and temperature dependence of the torque and magnetization of the complex.

## Conclusion

The combination of paramagnetic 3*d* and 4*f* ions in molecular units is a successful strategy to improve the single molecule magnet behaviour, mainly thanks to the reduction of tunnel efficiency. The design of better performing SMMs requires the optimization of the magnetic anisotropy of the individual ions and of their orientation. The previously encountered difficulties in the determination of the anisotropic contributions of this polynuclear molecule, evidence the need to use the combination of experimental tools and *ab initio* calculations to fully unravel the magnetic properties of such complex systems. Moreover, fragmentation of the molecular framework to simplify these calculations should be ruled out as much as possible to avoid spurious effects. The SCM technique allows us to work at low field, thus nicely complementing CTM that instead is more suitable to investigate the high field regime. The experimental results of our investigation unequivocally point to a more toroidal like orientation of the anisotropy axes of the Dy ions than previously predicted. However, the employed simplified phenomenological model reproduces the high field values of the magnetization but fails in reproducing the low field regime. This is not surprising since our model does not take into account the exchange interaction between all magnetic ions neither the low-lying excited Kramers doublets on the Dy sites. The work of improving the description of the field and temperature dependency of the magnetic anisotropy is currently ongoing.

## Data Availability Statement

The authors can provide the detailed employed basis set contractions upon simple demand.

## Author Contributions

JR performed the synthesis and crystallisation of the compounds. Crystal structure data were recorded and processed by CA. Single crystal orientation and SQUID measurements were realised by GC and M-EB. GC performed the simulations. Magnetic curves and susceptibility thermal variation measurements were realised by MP. Torque magnetometry was performed by M-EB and MP. MP performed the simulations. *ab initio* calculations where realised by XG and LU. RS, AP, and LC supervised the different parts of the work. All authors contributed to the discussion of the results. The manuscript was written by M-EB, MP, and LU. M-EB managed the project.

### Conflict of Interest Statement

The authors declare that the research was conducted in the absence of any commercial or financial relationships that could be construed as a potential conflict of interest.
