# Supplementary material for: Single Crystal Investigations Unravel the Magnetic Anisotropy of the “Square-In Square” Cr4Dy4 SMM Coordination Cluster
Source: Front Chem. 2019 Jan 24;7:6. doi: 10.3389/fchem.2019.00006 (PMC6353784; doi:10.3389/fchem.2019.00006)
Supplement: Supplementary file 1 [file Data_Sheet_1.PDF]

Supplementary Information:

Single crystal investigations unravel the individual anisotropy contributions to the SMM behaviour of the "Square-in Square" Cr_4Dy_4 coordination cluster

Mauro Perfetti, Julia Rinck, Giuseppe Cucinotta, Christopher E. Anson, Xuejun Gong, Liviu Ungur, Liviu Chibotaru, Marie-Emmanuelle Boulon, Annie K. Powell, Roberta Sessoli

Table S1: Direction of the rotation axis and the magnetic field at 0° for each experiment

Technique	Rotation axis direction	Magnetic field direction at 0°
SCM:	(0, 0, 1)	(1, 0, 0)
	(1, 0, 0)	(0, 0, 1)
DC magnetometry:	(0, 0, 1)	-
	(0.707, 0.707, 0)	-
CTM:	(0, 0, 1)	(1, 0, 0)
	(0.707, 0.707, 0)	(-0.707, 0.707, 0)

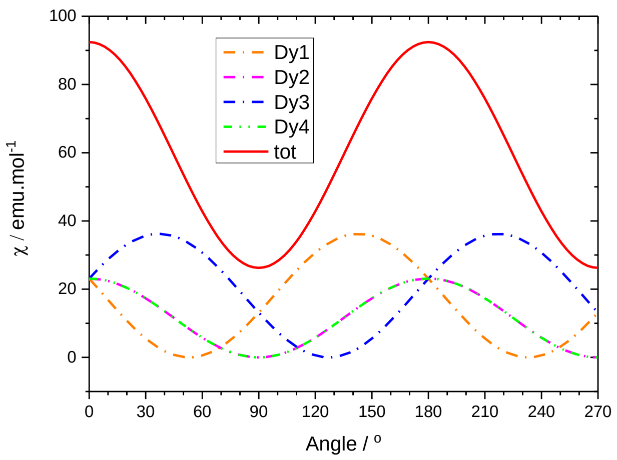

Figure S1: Simulation of the angular dependence of the magnetic susceptibility with previously proposed parameters. The coloured dashed lines are the single contributions while

the red continuous line is the sum of the four contributions. This simulation assumes no interactions and a factor g = 19.76.

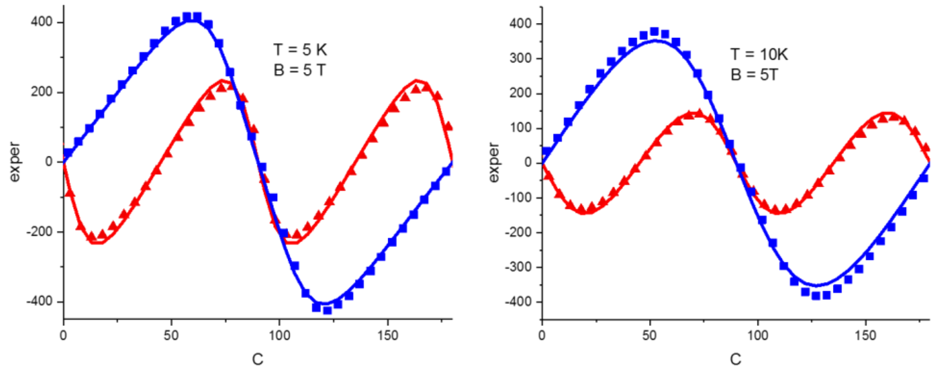

Figure S2: Experimental (symbols) and simulations (lines) of the CTM rotation, blue: out-of-plane, red: in-plane.

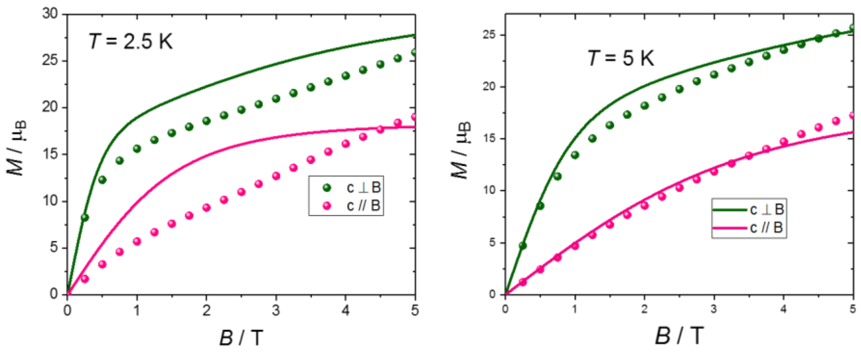

Figure S3: Simulation of the magnetization curves at 2.5 K and 5 K for magnetic field perpendicular (green) and parallel (pink) to the ***c*** directions

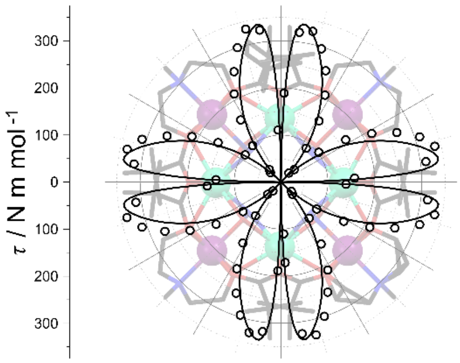

Figure S4: Polar plot of the torque moment superimposed to the molecular structure, at 2 K and 5 T; dots are the experimental points and the line is the simulation.
